# London Post-Graduate Institutions

**Published:** 1912-09-07

**Authors:** 


### LONDON POST-GRADUATE INSTITUTIONS.

### WEST LONDON POST-GRADUATE COLLEGE.

The West London Hospital, Hammersmith, was the first of the general hospitals in London to reserve its practice strictly for qualified men, and to establish a post-graduate College as an integral part of its constitution. The hospital itself contains 160 beds, and the advantages of the direct connection of the College with the wards and out-patient work are obvious. It is recognised by the Royal College of Surgeons as an institution where candidates (who need not be members) for the Fellowship can spend the necessary year of study after obtaining a registrable qualification, while the College and hospital are also recognised by the naval and military authorities as regards study leave. The hospital is authorised by the University of London to give certificates of post-graduate study for the M.D. and M.S degrees.

Members of the College may also accompany the resident staff on their visit to the wards, and so have opportunities of refreshing and extending their knowledge of clinical methods. Demonstrations are given each morning by the assistant physician and by the assistant surgeons. Operations are performed daily at 2.30 p.m. Post-graduates are allowed to stand near the table and can see the operations perfectly. The surgeons often avail themselves of the assistance of post-graduates at operations.

The out-patient departments necessarily resemble in their main features those of other general hospitals, and the staff are always alert to discover, and, when found, to demonstrate, any cases of exceptional interest, or, what is of equal importance, any points of special interest found in cases of the more common diseases. During the sessions lectures are given daily, except Saturdays, at 5 p.m., on practical medicine and surgery in all their branches. There are also short courses on tropical diseases, and on mental diseases by men who have made their mark in those subjects.

Small classes are formed, when required, in connection with diseases of special regions, such as eye, throat, skin, etc.; for the clinical examination of blood and urine; in vaccine therapy, bacteriology and medical microscopy generally; in applied anatomy, administration of anæsthetics, intestinal surgery, cystoscopy, $x$-ray work, etc. For these classes, each of which is strictly limited to a small number, additional fees are required, as they are practically individual and tutorial. The ordinary fee for membership covers attendance at all general lectures and at all the routine practice of the hospital, whether in the general or special wards or departments. Autopsies can, of course, be attended. Arrangements can be made through the College authorities for special coaching should it be desired.

Members may join for any period from one week upwards, the fees ranging from one guinea to £30.

### THE LONDON POLYCLINIC.

The Medical Graduates' College and Polyclinic, founded in 1899, mainly through the efforts of Mr. (now Sir) Jonathan Hutchinson, has now a well-recognised position among the medical educational organisations of the metropolis. Its object is to provide opportunities for clinical and practical study for those who have already gained a position on the medical register. From the outset two principles have been recognised as essential to a large and broad scheme of post-graduation study. First, that such a scheme should be entirely separate and distinct from the ordinary educational course of the medical student. Secondly, that the teaching should not be confined to the staff of any individual hospital, but should include capable and distinguished representatives of all schools. Hence, at the Polyclinic, the needs of the practitioner receive full consideration, and the teaching in a very special manner represents the work of many of the best-known physicians and surgeons in the metropolis.

The afternoon cliniques are conducted daily at four o'clock, and offer valuable opportunities. Medicine, surgery, and each of the more special branches of practice, has its appropriate day. Selected cases are demonstrated and made the subject of clinical comment, and opportunities for personal examination by those attending the clinique are offered. In this way, and in a comparatively short period of time, the practitioner is able to overtake a large quantity of interesting and instructive clinical material under the direction of teachers specially qualified in each of the various departments. Indeed, it is difficult to imagine any scheme more suitable for those who are anxious to increase their clinical experience both in general and special work. On four days of each week there is also a lecture, illustrated by lantern slides, pathological specimens, or clinical cases, by a teacher of repute from one of the provincial or metropolitan schools. In these lectures the practical note is maintained throughout.

With a view to render these opportunities accessible to all, the annual subscription, which includes admission to all cliniques and lectures and also the use of the library and museum has been fixed at one guinea. In addition, the *Polyclinic Journal* is issued monthly, and is sent free to all subscribers. In the College laboratory specimens are examined and reported on for small fees, and here practical personal instruction in all the branches of clinical pathology can be obtained. Another part of the Polyclinic scheme, and one of great importance, is the provision of "practical classes" in the more recent methods of clinical investigation. Included in this division, instruction is provided in the use of the ophthalmoscope, laryngoscope, otoscope, cystoscope, sigmoidoscope, and other specialised clinical instruments; in the clinical examination of the blood, sputa, urine, etc.; in the use of the $x$-rays; and in neurology, electro-therapeutics, orthopædics, intestinal surgery, and gynæcology. In each class the numbers are limited.

Tutorial classes for practitioners reading for the higher examinations have recently been instituted, and have proved most successful. Particulars as to fees, hours, etc., can be obtained upon application to the Medical Superintendent, 22 Chenies Street, Gower Street, W.C.

### THE LONDON SCHOOL OF CLINICAL MEDICINE (POST-GRADUATE).

This School, which is devoted exclusively to post-graduate study, has its headquarters at the "Dreadnought" Hospital, Greenwich. Affiliated to it for the purposes of teaching are the following institutions:—The Royal Waterloo Hospital for Children and Women, the Bethlem Royal Hospital, and the Miller General Hospital. At the "Dreadnought" Hospital there are in addition to the medical and surgical wards special wards for the treatment of patients suffering from venereal disease, the medical and surgical forms of tuberculosis, diseases of the eye, ear, nose, and throat. In addition there are unusual opportunities for practical instruction in operative surgery and pathology. Every variety of disease may be studied at the "Dreadnought" and the hospitals affiliated thereto. A series of lectures is given each session by members of the staff of the "Dreadnought," Emeritus lecturers and Extramural lecturers, and by special lecturers of eminence in this country and abroad. The syllabus containing full information may be obtained from the Dean, C. C. Choyce, at the "Dreadnought" Hospital, Greenwich.

### NORTH-EAST LONDON POST-GRADUATE COLLEGE.

This School, which is in connection with the Prince of Wales's General Hospital, Tottenham, N., is recognised by the University of London, the Admiralty, the India Office, and the University of Cambridge in regard to bacteriology for the D.P.H. diploma as a place of post-graduate study. Opportunities are here afforded for attending demonstrations on various branches of medicine, surgery, and gynæcology, diseases of the eye, ear, throat, nose, skin, fevers, diseases of children, psychological medicine, anæsthetics, $x$-rays, bacteriology, and dentistry. Special classes with a limited attendance have been arranged for these and other subjects. The fee for a three-months' course in any single department is one guinea, or three guineas admits for a similar term to the whole practice of the hospital. A perpetual ticket costs five guineas. Additional information, with syllabus of lectures and special classes, from the Dean.

